# Phenotypic Evaluation of Soybean Genotypes for Their Reaction to a Mississippi Isolate of *Phakopsora pachyrhizi* Causing Soybean Rust

**DOI:** 10.3390/plants12091797

**Published:** 2023-04-27

**Authors:** Shuxian Li, James R. Smith

**Affiliations:** United States Department of Agriculture, Agricultural Research Service (USDA, ARS), Crop Genetics Research Unit, 141 Experiment Station Road, Stoneville, MS 38776, USA

**Keywords:** soybean, soybean rust, *Phakopsora pachyrhizi*, resistance, breeding

## Abstract

Soybean rust (SBR) caused by *Phakopsora pachyrhizi* Syd. and P. Syd. is one of the most important foliar diseases of soybean. SBR has the potential to cause major economic damage to global and U.S. soybean production. Analysis of reactions of soybean genotypes to *P. pachyrhizi* is an important step towards breeding for resistance to SBR. Fifty-four diverse soybean genotypes with both known and unknown *Rpp* resistance genes were tested for their reactions to a Mississippi *P. pachyrhizi* isolate. PI 567102B (*Rpp6*) had a near-immune reaction with the lowest disease severity score and no sporulation. Among seventeen genotypes with resistant or incomplete resistant reddish-brown (RB) reactions, eight are improved breeding lines that are available to researchers through material transfer agreements (MTAs). Thirty-six genotypes had the susceptible TAN reaction. Four soybean lines (RN06-32-1(7-b, GC 00138-29, G01-PR16, and GC 84051-9-1) had RB reactions and significantly lower SBR severity and sporulation than three of the six resistant checks, PI 230970 (*Rpp2*), PI 462312 (*Rpp3*), and PI 459025B (*Rpp4*). G01-PR16 is a publicly released germplasm. This research provides new information about reactions of different soybean genotypes to a midsouthern USA isolate of *P. pachyrhizi* and thereby aids in breeding for resistance to SBR.

## 1. Introduction

Soybean rust (SBR) is one of the most economically important foliar diseases of soybean (*Glycine max* (L.) Merr.), occurring in many major soybean-producing countries. Significant soybean yield losses of up to 80% to 90% due to soybean rust have been reported in many countries, including Brazil and the U.S. [1,2]. These losses result primarily from a reduction in pods, seeds per pod, and seed weight [1] due to a decrease in photosynthesis in infected leaves and premature defoliation [3]. The soy market could be directly impacted by SBR. The reduction of grain due to SBR could lead to a decrease in the production of soy oil, protein, and related derivatives [4].

Soybean rust is caused by the obligate biotrophic fungal pathogen *Phakopsora pachyrhizi* Syd. and P. Syd. [5]. The pathogen was first described in Japan in 1902 by Hennings who named the pathogen *Uredo sojae* Henn. [6]. Later, Hans and Paul Sydow gave the fungus its current name, *Phakopsora pachyrhizi* Syd. and P. Syd, based on an isolate obtained from the leguminous host plant *Pachyrhizus erosus* (L.) Urb. (=*Pachyrhizus angulatus*) in Taiwan [7,8]. Since then, *P. pachyrhizi* has spread and can be found in many soybean-growing regions around the world. The first discovery of soybean rust in the United States was in Hawaii in 1994 [9]. The disease was first detected in the continental United States in a field near Baton Rouge, Louisiana on 6 November 2004 [10].

In general, soybean has three major types of reaction in response to *P. pachyrhizi* infection: (1) resistant immune (IM) reaction with no macroscopically visible lesions; (2) resistant or incomplete resistance with reddish-brown (RB) lesions; and (3) a susceptible reaction with tan (TAN) lesions [11,12]. The TAN lesion type is usually associated with high levels of sporulation of the pathogen, but two types of TAN lesions have been described: “few” or “many” uredinia per lesion [5]. The reddish-brown colored lesion type can be sporulating or non-sporulating, whereas the immune reaction is defined as a lack of visible lesions on leaves after being challenged with *P. pachyrhizi* [5,13]. The IM and RB reactions, with low sporulating or non-sporulating lesions, have been considered resistant reactions to the SBR causal pathogen.

Seven resistance genes (*Rpp 1–7*) to *P. pachyrhizi* [14,15,16,17,18,19,20,21,22] have been identified, but each of these *Rpp* genes conditions resistance to only a limited number of *P. pachyrhizi* isolates [23,24,25]. Identification of genotypic sources of resistance is the first step towards developing cultivars with resistance to soybean rust. Moreover, as no one *Rpp* gene has resistance to all isolates, there is a continuing need to identify new sources of soybean rust resistance.

Soybean [*Glycine max* (L.) Merr.] is one of the most important legume crops in the world. It is a major crop in Mississippi where it provides income to local U.S. economies and is an important source of high-quality plant protein and oil to the U.S. and the world [26,27]. Moreover, Mississippi was one of the first states in the USA where soybean rust was detected on soybean in Adams County on 16 November 2004 [28]. We hypothesized that a diverse group of soybean genotypes with both known and unknown *Rpp* resistance genes, including multiple improved breeding lines derived from the diverse sources, would have differential reactions to a domestic isolate of *P. pachyrhizi* from Mississippi. Soybean genotypes identified to have resistance to this southern *P. pachyrhizi* isolate could be utilized for the development of *Rpp* gene pyramids or crossed with other resistance resources to obtain more durable resistance to SBR and thereby aid in breeding for resistance to SBR.

Hence, the objectives of this research were to analyze the phenotypic reactions of soybean genotypes to SBR and determine their suitability for use in breeding programs. Rust lesion type, disease severity, and *P. pachyrhizi* sporulation for the genotypes were compared and analyzed. This research provides new information for the selection of breeding materials to aid in breeding resistance to SBR.

## 2. Results

Results of the analysis of variance (not shown) indicated that there werea significant (*p* ≤ 0.05) differences among soybean genotypes that were associated with the different sources of genotypic resistance.

### 2.1. Reaction/Lesion Type

**Figure 1:**
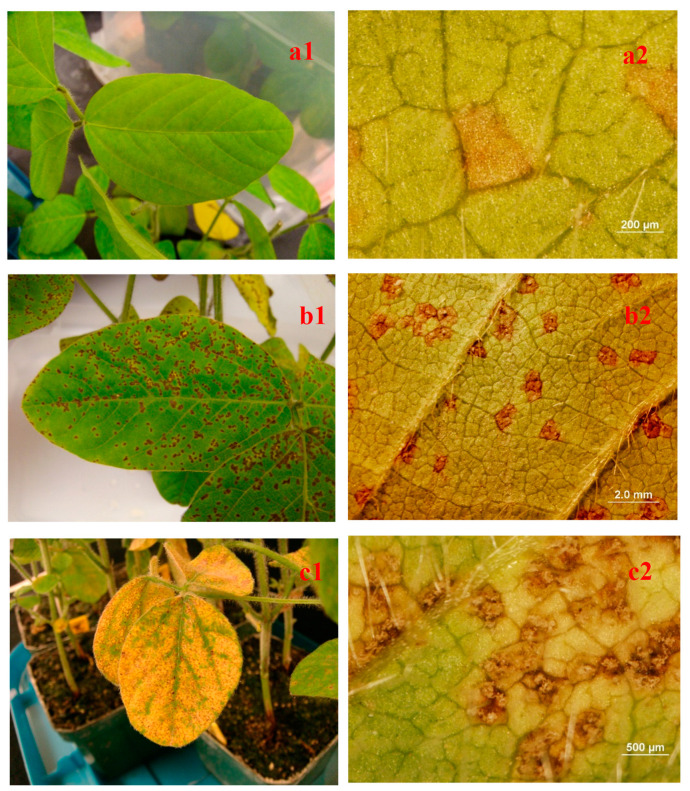
Reactions of soybean genotypes to a Mississippi isolate MS 10-6b of *Phakopsora pachyrhizi* 14 days after inoculation on 21-day-old soybean seedlings at the V2–V3 growth stage. a. Near-immune reaction on PI 567102B (*Rpp6*). (**a1**): Leaf, (**a2**): lesion without sporulation; b. reddish-brown (RB) reaction on PI 230970 (*Rpp*2). (**b1**): Leaf symptoms, (**b2**): lesions with or without sporulation; c. tan-colored (TAN) reaction on soybean cultivar Williams 82. (**c1**): Leaf symptoms, (**c2**): lesions with profuse sporulation.

Of the 54 soybean genotypes tested (Table 1), one genotype, PI 567102B (*Rpp6*), had a near-immune reaction after inoculation with the *P. pachyrhizi* isolate MS06-1b (Table 2). Without magnification, none of the plants had visible lesions. Under the dissecting microscope, 4 out of 5 plants were not found with lesions (Severity = 1), and 1 had a few small lesions (severity = 2) with an average of 1.2. (Figure 1(a1,a2)). Among the 17 genotypes with RB reactions (Table 2, Figure 1(b1,b2)), 3 were released germplasm lines (CM422, DS5-67, and G01-PR16), 3 were the GC lines from the World Vegetable Center (WVC), and 6 were improved breeding lines (unreleased) that were derived from *Rpp2*-resistant PI 230970 (4013-2-1-1-4-1 and 4014-242-341), *Rpp4*-resistant PI 459025B (4022-1-4-124, and 4022-1-4-121), *rpp3*-resistant PI 567099A (7092-x-1), and PI 605891A (RN06-32-1(7-b). Included in the 17 with RB lesions were 5 resistant checks with known resistance genes, which were PI 200492 (*Rpp1a*), PI 230970 (*Rpp2*), PI 462312 (*Rpp3*), PI 459025B (*Rpp4*), and PI 200487 (*Rpp5*). In contrast, 36 genotypes, including all susceptible checks (Williams 82, Stafford, 5002T, Osage, NC-Roy), had the susceptible TAN reaction (Table 2, Figure 1(c1,c2)). Included in the 36 genotypes with TAN lesions were PI accessions and breeding lines with resistance genes allelic to the *Rpp1a* gene *(Rpp1b*, PI 594538A, RN6-313-362, and RN6-3-134211; *Rpp1c*, PI 587880A, 6108-1043321, and 6108-1662111; *Rpp1d*, PI 587886, 6112-13-6211, 6112-55-3-1-21, 6112-13-3-1-11, 6112-13-6-42, 6112-13-33-1-1, and 6112-136-411; and *Rpp1e*, PI 587905 and 6113-216-121). Multiple PI accessions and breeding lines with unmapped resistance gene(s) were also susceptible to the Mississippi isolate including PI 605779E and its derived line 6116-5-4-1-1; PI 567145C and its derived lines 88-203-1-2-1, 7088-198-212, and 7088-198-521; PI 594723 and its derived line 6115-22-511; and PIs 594760B and 605833. PI 200456 (*rpp5*) and PI 567099A (*rpp3*) with known mapped resistance genes also had TAN lesions (Table 2). The case of PI 567099A is interesting because its derived line, 7092-x-1, had an RB reaction. The case of breeding line 6106-132-1-2 with a TAN reaction in this study is also interesting because it is derived from PI 567102B, which had a near immune response in this study. The reactions of three other improved breeding lines also provided insightful data. Although derived from parents (L86-1752 derived from PI 230970 with *Rpp2* and GC 00138-29) that both have resistance to MS06-1b, breeding line 4013-1-3-5-3-2 had a TAN reaction to MS06-1b. Likewise, breeding lines 4018-4-1-1-212 and 4019-2-2-3-3-1 had TAN reactions in this study even though both parents (L87-0482 derived from PI 459025B with *Rpp4* and GC 84051-9-1) were resistant to MS06-1b.

### 2.2. Rust Severity

Statistically, there were significant (*p* ≤ 0.05) differences in soybean rust severity among the soybean genotypes tested. The mean soybean rust severity of all genotypes tested was 3.5 with a range of 1.2 to 4.7 based on a lesion density scale of “1”, which was no visible lesions, to “5”, which was the highest score of disease severity that featured prolific lesion development covering most of the leaf [29,30]. PI 567102B (*Rpp6*) had the lowest rust severity score of 1.2. Among 17 genotypes with RB reactions, 11 had severity scores of less than 3, where PI 200492 (*Rpp1a*) and PI 200487 (*Rpp5*) had low rust severity scores of 1.3 and 1.9, respectively. In contrast, Williams 82, NC-Roy, and PI 587905 (*Rpp1e*) had high severity scores of 4.6, 4.6, and 4.7, respectively (Table 2). Rust severity analysis was also performed based on lesion type (RB and TAN). Differences in the severity scores among soybean genotypes producing an RB reaction type were significant (*p* ≤ 0.05). Among them, four lines with RB reactions (RN06-32-1(7-b, GC00138-29, GC84051-9-1, and GC84051-9-1) had significantly lower SBR severity than three of the six resistant checks, PI 230970 (*Rpp2*), PI 462312 (*Rpp3*), and PI 459025B (*Rpp4*). The severity scores for the RB group ranged from 1.3 to 3.6 with a mean of 2.8 (Table 2). Among genotypes with the TAN lesion type, there were also significant (*p* ≤ 0.05) differences for soybean rust severity. The severity scores for the TAN group ranged from 3.1 to 4.7 with a mean of 4.0 (Table 2), which was higher than the mean severity of the RB group (2.8). The lower end of the TAN range for severity (3.1) overlaps with the upper end of the severity range (3.6) for the RB group.

### 2.3. Rust Sporulation

Soybean rust sporulation differed significantly (*p* ≤ 0.05) among soybean genotypes. The mean sporulation score was 3.6 with a range from 1 to 4.9 based on the 1–5 scale where 1 refers to no sporulation on lesions, and 5 indicated over 75% of the lesions were sporulating [30,31]. No sporulation was observed on inoculated soybean leaves of PI 567102B (*Rpp6*) (Table 2, Figure 1(a2)). Among the 17 genotypes with RB reactions, 10 had sporulation scores of less than 2. Eight genotypes (G01-PR16, GC00138-29, RN06-32-1(7-b, GC84051-9-1, 7092-x-1, DS5-67, 4013-2-1-1-4-1, and 4022-1-4-124) also had significantly (*p* ≤ 0.05) lower sporulation ratings than the resistance checks PI 230970 (*Rpp2*) and PI 459025B (*Rpp4*). Susceptible soybean Williams 82 and breeding line 6108-1043321 had sporulation scores of 4.8 and 4.9, respectively (Table 2). For genotypes producing RB reactions, the mean sporulation was 2.3 with a range from 1.2 to 3.6, whereas for genotypes with TAN lesions, the mean sporulation was 4.4 with a range from 3.3 to 4.9 (Table 2). Just as for severity, the mean sporulation for the RB group was less than that of the TAN group, and the ranges of the two groups overlapped.

### 2.4. Correlation Analysis between Soybean Rust Severity and Sporulation

**Figure 2:**
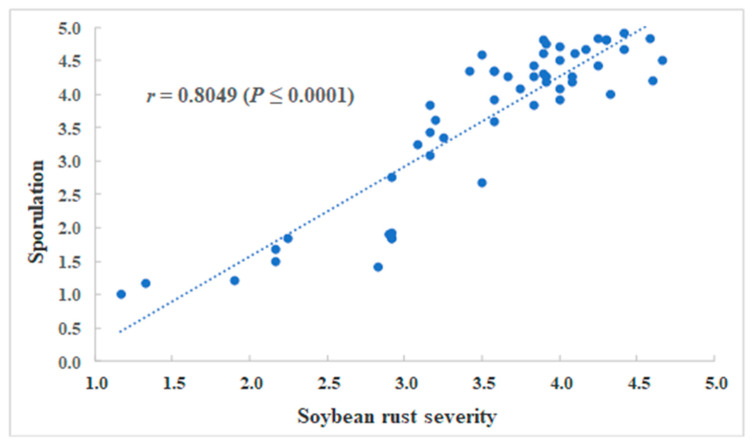
Correlation between soybean rust severity and sporulation of 54 soybean genotypes evaluated against a Mississippi isolate MS 10-6b of *Phakopsora pachyrhizi*. Soybean rust severity was evaluated using a 5-point scale based on lesion density of the percentage of infected area where 1 = no visible lesions, 2 = a few lesions (1–20% infected area), 3 = moderate lesion density (21–50% infected area), 4 = heavy lesion density (51–80% infected area), and 5 = very heavy lesion density over most of the leaf (81–100% infected area). Sporulation of *P. pachyrhizi* was rated based on the relative percentage of lesions producing urediniospores on each plant tested using a 1-to-5 scale where 1 = no sporulation, 2 = less than 25%, 3 = 26 to 50%, 4 = 51 to 75%, and 5 = 76 to 100% of the lesion sporulating [29,30,31].

Results indicated that soybean rust severity over all genotypes tested was significantly correlated with soybean rust sporulation (*r* = 0.8049, *p* ≤ 0.0001), (Figure 2). For soybean genotypes with RB lesion types, soybean rust severity was positively correlated with soybean rust sporulation (*r* = 0.653, *p* ≤ 0.0001). For soybean genotypes producing TAN lesions, soybean rust severity was also significantly (*r* = 0.3656, *p* ≤ 0.0001) correlated with soybean rust sporulation.

## 3. Discussion

Soybean rust is one of the most economically important soybean foliar diseases [32,33,34]. Although yield losses have not been significant in the United States since the first discovery of soybean rust in the continental U.S. in 2004 [10], the disease still has the potential to cause major economic damage to U.S. soybean production. This is because the causal agent *P. pachyrhizi* has a broad host range [35], is airborne [36], and has a diverse genetic structure [37,38], complex virulence patterns, and a high level of pathotype diversity [15,38,39,40,41]. The broad host range of the pathogen has facilitated the spread of *P. pachyrhizi*. The ability of *P. pachyrhizi* urediniospores to be spread by the wind for long distances increases the opportunity for widespread distribution worldwide and has the potential for causing severe yield losses in any soybean growing area [42,43]. Moreover, the possibility of continuing adaptation of the pathogen in the continental U.S. should not be underestimated. Currently, no soybean cultivars grown in the U.S. are reported to be resistant to all isolates of *P. pachyrhizi*. When weather conditions are favorable for disease development, serious epidemics could occur. Therefore, developing soybean cultivars with resistance to domestic isolates of *P. pachyrhizi* is needed to combat emerging potential threats of soybean rust in the U.S. Analysis of reactions of soybean genotypes to *P. pachyrhizi* is an important step towards breeding for resistance to SBR.

The USDA Soybean Germplasm Collection located at the University of Illinois (Urbana, IL) maintains over 21,810 accessions of the genus *Glycine* with over 19,626 plant introductions from 92 countries (https://www.ars-grin.gov/npgs/, accessed on 16 February 2023). The accessions are a collection of natural genetic diversity. Miles et al. [29] screened 16,595 soybean accessions from the USDA Germplasm Collection with 4 foreign *P. pachyrhizi* isolates from Brazil, Paraguay, Thailand, and Zimbabwe at the USDA, ARS, Foreign Disease-Weed Science Research Unit located in Maryland. Some potential SBR-resistant accessions that had low rust severities with an RB-resistant reaction were identified. In another study, Walker et al. [44] evaluated 118 soybean germplasm accessions for resistance to SBR at up to 5 locations in the southern United States. No accession was immune to soybean rust in all field trials at the five locations.

To identify new sources of resistance to domestic *P. pachyrhizi* isolates, one of our strategies was to use domestic isolates to evaluate the soybean lines that were previously identified as resistant to foreign isolates [30,45]. In our previous study, the 10 plant introductions that were reported as resistant in Paraguay [46] were selected and tested using *P. pachyrhizi* isolates from Mississippi [30]. This approach resulted in the successful identification of new sources of resistance to American and Paraguayan isolates of *P. pachyrhizi*, which led to the identification and mapping of the *Rpp6* gene in PI 567102B and PI 567104 [19,20].

Resistance to soybean rust has often been identified primarily based on infection/lesion types. However, variations in the RB and TAN reaction types have been reported [12,25,30]. Not all rust lesion types are typical in relation to levels of severity and sporulation. It has been suggested that the RB and TAN reaction types should be subdivided based on the levels of sporulation [5]. An assessment of the resistance to soybean rust was conducted not only for lesion color but also for uredinia of *P. pachyrhizi* per lesion, the frequency of lesions that had uredia, and the frequency of open uredinia and sporulation level [47]. In another study, Paul et al. [25] found that lesion color was usually a reliable indicator of the average number of uredinia per lesion and that using lesion color to classify infection type could be much quicker than counting uredinia per lesion [25].

In our present study, lesion type was not strongly predictive of rust severity and sporulation level, although the mean severity and sporulation for RB lesion types were lower than those for TAN lesions. For example, the RB-classified breeding line 4014-242-341 had scores of 3.6 for both rust severity and sporulation, which were relatively high, whereas the mean rust severity and sporulation scores for all RB-classified genotypes were only 2.8 and 2.3, respectively. In contrast, PI 567099A produced TAN lesions, but its severity score was only 3.1, which was lower than that of six RB genotypes, including the resistant check PI 459025B (*Rpp4*). Breeding line 7092-x-1 derived from PI 567099A had an RB reaction type unlike its parent but had a similarly low severity score (2.9) as its parent. However, the sporulation score of 7092-x-1 was significantly lower (1.8) than that of its parent (3.3). The cause for the more resistant lesion type and lower sporulation level in the progeny (7092-x-1) versus the parent (PI 567099A) is uncertain. It is possible that 7092-x-1 inherited a more favorable genetic background from its other parent (LG01-5087-9) for a fuller expression of resistance of the *rpp3* gene derived from PI 567099A. This would be an example of epistasis where the *rpp3* gene expresses a more resistant phenotype in the progeny compared to the parent because of its differential interaction with the genotypic background of the progeny versus the parent. The genetic mechanisms of the *Rpp* genes and their interactions with diverse genotypic backgrounds may not be fully understood and need further investigation.

Multiple soybean lines with different resistance alleles at the *Rpp1* locus on Chr 18 were tested against the Mississippi isolate of *P. pachyrhizi* (MS 06-1b) in this study. Interestingly, all soybean lines containing either *Rpp1b*, *Rpp1c, Rpp1d,* or *Rpp1e* had TAN lesions and were susceptible to the Mississippi isolate. This result was in agreement with the findings from another study [25]. It was reported that the “*Rpp1b* gene of PI 594538A and the *Rpp1* allele in PI 587880A had not provided any resistance to *P. pachyrhizi* populations in the U.S.” However, all of these lines were effective against some *P. pachyrhizi* isolates and populations in South America [48,49]. PI 200492 with the *Rpp1a* allele at the *Rpp1* locus had a susceptible reaction to some of the U.S. *P. pachyrhizi* isolates [25], but in our study, PI 200492 had an RB-resistant reaction against the Mississippi isolate with low severity and sporulation scores of 1.3 and 1.2, respectively.

Soybean breeding line 6106-132-1-2 had a TAN reaction even though it was developed from DS-880 x PI 567102B. Although one of its parents (PI 567102B with *Rpp6*) had the highest level of resistance against the Mississippi isolate, 6106-132-1-2 had high severity (3.9) and sporulation (4.2) scores to go with its TAN lesion type. As 6106-132-1-2 did not inherit *Rpp6* from PI 567102B but still had RB reaction types to multiple isolates used by Stone et al. [49], it likely has a resistance gene that is different from *Rpp6* but was still likely inherited from PI 567102B, as DS-880 has no known rust resistance.

Breeding line 4013-1-3-5-3-2 had a TAN reaction against MS06-1b in this study, but RB reactions to other isolates used by Stone et al. [49]. The resistance in 4013-1-3-5-3-2 was likely derived from GC 00138-29, as L86-1752 likely has only *Rpp2*. However, as GC 00138-29 was resistant in the current study and 4013-1-3-5-3-2 was not, it is likely that GC 00138-29 has multiple resistance genes and that 4013-1-3-5-3-2 inherited one that did not provide resistance to MS06-1b, but it did provide resistance to the isolates used by Stone et al. [49]. Likewise, breeding lines 4018-4-1-1-212 and 4019-2-2-3-3-1 had TAN reactions in this study and therefore did not inherit *Rpp4* from L87-0482 but must have derived the resistance observed by Stone et al. [49] from their other parents, GC 84051-9-1 and GC 00138-29, respectively, which each likely has multiple resistance genes and both were resistant in the current study.

Different levels of resistance were found among 54 soybean genotypes to a Mississippi isolate of *P. pachyrhizi* in our present study. Although none of the soybean lines showed completed immunity, PI 567102B containing the *Rpp*6 gene had a near-immune reaction with only a few lesions on some leaves but no sporulation. PI 567102B was also resistant to four of five *P. pachyrhizi* isolates from Alabama, Florida, and Louisiana [25] and was resistant to 12 of 16 isolates used by Stone et al. [49]. PI 567102B could be useful for the development of unique *Rpp* gene pyramids to obtain more durable resistance to SBR.

We identified four soybean lines (RN06-32-1(7-b, GC 00138-29, G01-PR16, and GC 84051-9-1) that had RB reactions and significantly lower SBR severity and sporulation than three of the six resistant checks, PI 230970 (*Rpp2*), PI 462312 (*Rpp3*), and PI 459025B (*Rpp4*). Further, eight lines (G01-PR16, GC00138-29, RN06-32-1(7b, GC 84051-9-1, 7092-x-1, DS5-67, 4013-2-1-1-4-1, and 4022-1-4-124) with RB reactions had significantly (*p* ≤ 0.05) lower sporulation ratings than PI 230970 (*Rpp2*) and PI 459025B (*Rpp4*). G01-PR16 (developed by the University of Georgia) and DS5-67 are already released and are freely available from the USDA collection. Breeding line 4022-1-4-124 is a sister line of CM422, both with *Rpp4*, but CM422 is already released and freely available from the collection. GC 00138-29, GC 84058-18-4, and GC 84051-9-1 are available from the WVC, and 4013-2-1-1-4-1 with GC 00138-29 in its pedigree is available by material transfer agreement (MTA) from the authors. Breeding line 4014-242-341 (RB lesion type in the current study) has GC 84058-18-4 in its pedigree, was resistant to all 16 isolates used by Stone et al. [49], and is available by MTA from the authors. Breeding line 4018-4-1-1-212 (TAN lesion type in the current study) has GC 84051-9-1 in its pedigree, was resistant to all 16 isolates used by Stone et al. [49], and is also available by MTA from the authors. Breeding line 7092-x-1 is available from the authors by MTA. These soybean lines are important sources for developing elite cultivars with broad resistance to SBR.

Considering the diverse genetic structure of the pathogen populations and pathogenic/virulence variations among isolates, experiments are currently underway to test selected soybean breeding lines against multiple *P. pachyrhizi* isolates from the U.S., with the goal to develop durable resistance to soybean rust and make it available to researchers.

## 4. Materials and Methods

### 4.1. Plant Materials and Breeding Line Development

Experiments were conducted at the USDA, ARS Jamie Whitten Delta States Research Center in Stoneville, Mississippi. Fifty-four soybean genotypes consisting of sixteen plant introductions (PIs) with both known and unknown *Rpp* resistance genes, three improved GC lines from the World Vegetable Center (WVC) (formerly the Asian Vegetable Research and Development Center (AVRDC)) in Taiwan, twenty-six unreleased improved breeding lines derived from the PI and GC sources, the six original resistance sources for the *Rpp* genes [PI 200492 (*Rpp1a*), PI 230970 (*Rpp2*), PI 462312 (*Rpp3*), PI459025B (*Rpp4*), PI 200487 (*Rpp5*), and PI 567102B (*Rpp6*)], five susceptible cultivar checks (Williams 82, Stafford, 5002T, Osage, and NC-Roy), and three improved released breeding lines [CM422 (PI 675076), DS5-67 (PI 698651), and G01-PR16 (PI 659503)] were used in this study. The soybean entry code, line name, pedigree or accession name, genotype, and maturity group information are listed in Table 1. Soybean seeds of the PIs, G01-PR16, and cultivars were obtained from the USDA Soybean Germplasm Collection in Urbana, IL and were increased at Stoneville, Mississippi, and Isabela, Puerto Rico. Seed of the GC lines was obtained from the World Vegetable Center and increased in Stoneville. The 26 unreleased improved breeding lines, along with CM422 and DS5-67, were developed and tested by USDA scientists at multiple locations and in collaboration with researchers in Paraguay from the Instituto Paraguayo de Tecnologia Agraria (IPTA) and the Camara Paraguayo de Exportadores de Cereales y Oleaginosas (CAPECO).

### 4.2. Pathogen Isolate, Purification, and Maintenance

A Mississippi *P. pachyrhizi* isolate (MS 06-1b) was used to test the reaction of all soybean genotypes. This pathogen was collected from urediniospores of naturally infected kudzu leaves in Jefferson County, Mississippi in August 2006. Three methods, including microscopy determination, enzyme-linked immunosorbent assay (ELISA), and polymerase chain reaction (PCR), were used to confirm the identity of the pathogen as previously described [28]. Increasing urediniospores were conducted by inoculating on leaves of a susceptible soybean cultivar Williams 82 grown in a growth chamber. The isolate was then purified as previously described [30]. Briefly, under an Olympus SZX12 dissecting microscope, a single uredinium from a lesion was picked with a small needle and reinoculated onto a leaf of Williams 82. After four such “inoculation–isolation” cycles, urediniospores from single-uredinium, isolate-infected leaves were collected using a Cyclone Surface Sampler (Burkard Manufacturing Co. Ltd., Rickmansworth, UK) connected to a vacuum pump. It was performed from 10 to 28 days after inoculation. Urediniospores and infected leaves were stored in a −80 °C freezer for further use.

### 4.3. Inoculum Preparation and Plant Inoculation

Inoculum was prepared using freshly collected urediniospores of MS 06-1b from Williams 82 plants in a growth chamber. As previously described [30], urediniospores were suspended in a solution containing 0.01% Tween-20 (vol/vol) in sterile distilled water and then filtered through a 100 mm cell strainer (BD Biosciences, Bedford, MA, USA) to remove clumped urediniospores and debris. Prior to inoculation, quantification of urediniospores in the suspension was determined using a hemocytometer and then diluted to a final concentration of 4 × 10^4^ urediniospores/mL.

Soybean entries were arranged in a randomized complete block design with two replications. Two seeds of each of the soybean lines were sown in each Jiffy poly-pak pot (Hummert, St. Louis, MO, USA). Plants in a pot were thinned to one plant per pot before inoculation. Sun Grow Metro Mix 360 soil (Sun Grow Horticulture Products, Belleview, WA, USA) was used to fill the pots. Pots were placed in a Conviron growth chamber under a 16 h photoperiod with a light intensity of 433 μEm^−2^ s^−1^ at 25 ± 2 °C. Plants were watered daily.

Inoculation was performed on 21-day-old seedlings at the V2–V3 growth stage [50] using a Preval sprayer (Younkers, NY, USA) at a rate of 1 mL of spore suspension per plant. A mock inoculation was carried out on two pots of Williams 82 plants to monitor infection of the same solution without urediniospores. After inoculation, plants were moved to a dew chamber in the dark at 22 °C overnight (approximately 16–18 h) and then placed back into the Conviron growth chambers where temperatures were maintained at 23 °C during the day and 20 °C at night under a 16 h photoperiod with a light intensity of 280 μEm^−2^ s^−1^. The same experiment was performed three times.

### 4.4. Assessment of Lesion Types, Rust Severity, and Sporulation

The lesion types, soybean rust severity, and sporulation of *P. pachyrhizi* on lesions were assessed 14 days after inoculation (DAI). Rust severity was determined based on lesion density (percentage of infected area) on the first trifoliate leaves. A 5-point scale was used as previously described [29,30] where 1 = no visible lesions, 2 = a few lesions with approximately 1–20% of the area infected, 3 = lesion density with approximately 21–50% of the area infected, 4 = substantial lesion density with approximately 51–80% of the area infected, and 5 = very heavy lesion density with approximately 81–100% of the area infected. Lesion types on each soybean line were also recorded and classified as “TAN”, “RB”, or immune reaction as previously described [5,13]. The “TAN” lesion type had tan-colored lesions and was considered a susceptible reaction, whereas the “RB” type lesion exhibited reddish-brown colored lesions and was considered a resistant or partial/incomplete resistant reaction [12]. An immune reaction indicated a lack of obvious symptoms. Sporulation of *P. pachyrhizi* was rated based on the relative percentage of lesions producing urediniospores on each plant tested using a 1-to-5 scale where 1 = no sporulation, 2 = less than 25% sporulation, 3 = 26 to 50% sporulation, 4 = 51–75% sporulation, and 5 = 76 to 100% of the lesions sporulating [30,31].

### 4.5. Data Analysis

Data were analyzed statistically using the general linear mixed model procedure (PROC Glimmix) in SAS [version 9.1, SAS Institute, Cary, NC, USA]. Means of soybean rust severity and sporulation scores of soybean lines were compared with Fisher’s protected least significant difference (LSD) at *p* ≤ 0.05 unless otherwise stated. A Pearson correlation coefficient analysis of soybean rust severity and sporulation was performed on rating scores from all genotypes, as well as separately on the soybean lines producing either RB or TAN reactions.

## 5. Conclusions

This research provides new information about the reactions of different soybean genotypes to a midsouthern USA isolate of *P. pachyrhizi***.** PI 567102B (*Rpp6*) had a near-immune reaction with the lowest disease severity score and no sporulation. Four soybean lines (RN06-32-1(7-b, GC 00138-29, G01-PR16, and GC 84051-9-1) had RB reactions and significantly lower SBR severity and sporulation than three of the six resistant checks, PI 230970 (*Rpp2*), PI 462312 (*Rpp3*), and PI 459025B (*Rpp4*). Those soybean lines could be utilized for the development of *Rpp* gene pyramids or crossed with other resistance resources to obtain more durable resistance to SBR and thereby aid in breeding for resistance to SBR.

## Figures and Tables

**Table 1:** A list of soybean genotypes evaluated for their reaction to *Phakopsora pachyrhizi* isolate MS 06-1b.

Entry Code	Line Name	Genotype	MG ^a^	Pedigree or Accession Name
SB01	CM422	*Rpp4*	V	5601T × L87-0482 (PI 547879)
SB02	4013-2-1-1-4-1	*Rpp2 &/or ?* ^b^	III	L86-1752 (PI 547878) × GC 00138-29
SB03	4014-242-341	*Rpp2 &/or ?* ^b^	III	L86-1752 (PI 547878) × GC 84058-18-4
SB04	6112-13-6211	*Rpp1d* ^c^	III	LG01-5087-101 × PI 587886
SB05	6112-55-3-1-21	*Rpp1d* ^c^	IV	LG01-5087-101 × PI 587886
SB06	6112-13-3-1-11	*Rpp1d* ^c^	III	LG01-5087-101 × PI 587886
SB07	RN6-313-362	*Rpp1b*	IV	NE2701 × PI 594538A
SB08	4013-1-3-5-3-2	Unknown	III	L86-1752 (PI 547878) × GC 00138-29
SB09	4018-4-1-1-212	Unknown	III	L87-0482 (PI 547879) × GC 84051-9-1
SB10	4019-2-2-3-3-1	Unknown	IV	L87-0482 (PI 547879) × GC 00138-29
SB11	6112-13-6-42	*Rpp1d* ^c^	IV	LG01-5087-101 × PI 587886
SB12	6112-13-33-1-1	*Rpp1d* ^c^	IV	LG01-5087-101 × PI 587886
SB13	6112-136-411	*Rpp1d* ^c^	IV	LG01-5087-101 × PI 587886
SB14	RN6-3-134211	*Rpp1b*	IV	NE2701 × PI 594538A
SB15	6108-1043321	*Rpp1c* ^c^	IV	JTN-5503 × PI 587880A
SB16	6116-5-4-1-1	Unknown	IV	DS-880 × PI 605779E
SB17	7092-x-1	*rpp3*	IV	LG01-5087-9 × PI 567099A
SB18	88-203-1-2-1	Unknown	V	DS-880 × PI 567145C
SB19	7088-198-212	Unknown	V	DS-880 × PI 567145C
SB20	7088-198-521	Unknown	V	DS-880 × PI 567145C
SB21	6108-1662111	*Rpp1c* ^c^	V	JTN-5503 × PI 587880A
SB22	6115-22-511	Unknown	V	DS-880 × PI 594723
SB23	6113-216-121	*Rpp1e* ^c^	VI	DS-880 × PI 587905
SB24	RN06-32-1(7-b	Unknown	V	Dillon × PI 605891A
SB25	6106-132-1-2	Unknown	IV	DS-880 × PI 567102B
SB26	DS5-67	*Rpp3*	V	Williams 82 × Ankur (PI 462312)
SB27	G01-PR16	*Rpp3 + Rpp5*	V	Dillon × Hyuuga
SB28	4022-1-4-124	*Rpp4*	V	5601T × L87-0482 (PI 547879)
SB29	4022-1-4-121	*Rpp4*	V	5601T × L87-0482 (PI 547879)
SB30	Williams 82	Susceptible	III	Williams (7) × Kingwa
SB31	PI 594538A	*Rpp1b*	VIII	‘Min hou bai sha wan dou’
SB32	PI 200492	*Rpp1a*	VII	Komata
SB33	PI 587886	*Rpp1d* ^c^	VI	Bai dou
SB34	PI 587880A	*Rpp1c* ^c^	VI	Huang dou
SB36	PI 587905	*Rpp1e* ^c^	VII	Xiao huang dou
SB37	PI 567099A	*rpp3*	VIII	MARIF 2740
SB38	PI 462312	*Rpp3*	VIII	Ankur
SB39	GC00138-29	Unknown	VI	Unknown
SB40	GC84058-18-4	Unknown	V	Unknown
SB41	GC84051-9-1	Unknown	IV	Unknown
SB42	PI 567102B	*Rpp6*	VII	MARIF 2767
SB43	PI 594723	Unknown	VII	He xian hei dou
SB44	PI 605779E	Unknown	VIII	Sample 42
SB45	PI 230970	*Rpp2*	VII	No. 3
SB46	PI 459025B	*Rpp4*	VIII	Bing nan
SB47	PI 594760B	Unknown	VIII	‘Gou jiao huang dou’
SB48	PI 567145C	Unknown	VIII	MARIF 2816
SB49	Stafford	Susceptible	IV	V66-318 × V68-2331
SB50	5002T	Susceptible	V	Holladay × Manokin
SB51	PI 605833	Unknown	IX	Sample 102
SB52	Osage	Susceptible	V	Hartz 5545 × KS 4895
SB54	NC-Roy	Susceptible	VI	Holladay × Brim
SB55	PI 200456	*rpp5*	VIII	Awashima Zairai
SB56	PI 200487	*Rpp5*	VIII	Kinoshita

^a^ Maturity group. ^b^ *Rpp2* and/or other gene(s). ^c^ Mapped to *Rpp1* but is not the same allele as *Rpp1b*.

**Table 2:** Reaction of soybean genotypes to *Phakopsora pachyrhizi* isolate MS 06-1b.

Entry Code	Line Name	Genotype	MG ^a^	Lesion Type ^b^	Severity ^c^		Sporulation ^d^	
SB42	PI 567102B ^e^	*Rpp6*	VII	NIM	1.2	p ^f^	1.0	r ^f^
SB01	CM422	*Rpp4*	V	RB	3.2	lmn	3.4	klm
SB02	4013-2-1-1-4-1	*Rpp2 &/or ?* ^g^	III	RB	2.9	n	1.9	o
SB03	4014-242-341	*Rpp2 &/or ?* ^g^	III	RB	3.6	hijkl	3.6	jkl
SB17	7092-x-1	*rpp3*	IV	RB	2.9	n	1.8	op
SB24	RN06-32-1(7-b)	Unknown	V	RB	2.2	o	1.7	op
SB26	DS5-67	*Rpp3*	V	RB	2.9	n	1.8	op
SB27	G01-PR16	*Rpp3 + Rpp5*	V	RB	2.8	n	1.4	pqr
SB28	4022-1-4-124	*Rpp4*	V	RB	3.5	ijklm	2.7	n
SB29	4022-1-4-121	*Rpp4*	V	RB	3.2	klmn	3.6	jkl
SB32	PI 200492 ^e^	*Rpp1a*	VII	RB	1.3	p	1.2	qr
SB38	PI 462312 ^e^	*Rpp3*	VIII	RB	2.9	n	1.9	o
SB39	GC00138-29	Unknown	VI	RB	2.2	o	1.5	opq
SB40	GC84058-18-4	Unknown	V	RB	3.2	lmn	3.1	mn
SB41	GC84051-9-1	Unknown	IV	RB	2.3	o	1.8	op
SB45	PI 230970 ^e^	*Rpp2*	VII	RB	2.9	n	2.8	n
SB46	PI 459025B ^e^	*Rpp4*	VIII	RB	3.3	klmn	3.3	lm
SB56	PI 200487 ^e^	*Rpp5*	VIII	RB	1.9	o	1.2	qr
SB04	6112-13-6211	*Rpp1d* ^h^	III	TAN	4.0	cdefgh	4.1	fghi
SB05	6112-55-3-1-21	*Rpp1d* ^h^	IV	TAN	4.0	cdefgh	3.9	hij
SB06	6112-13-3-1-11	*Rpp1d* ^h^	III	TAN	3.6	hijkl	4.3	cdefgh
SB07	RN6-313-362	*Rpp1b*	IV	TAN	4.1	cdefg	4.3	defghi
SB08	4013-1-3-5-3-2	Unknown	III	TAN	3.6	hijkl	3.9	hij
SB09	4018-4-1-1-212	Unknown	III	TAN	3.8	efghij	4.3	defghi
SB10	4019-2-2-3-3-1	Unknown	IV	TAN	3.9	defghij	4.6	abcde
SB11	6112-13-6-42	*Rpp1d* ^h^	IV	TAN	3.4	jklm	4.3	cdefgh
SB12	6112-13-33-1-1	*Rpp1d* ^h^	IV	TAN	3.6	hijkl	4.3	cdefgh
SB13	6112-136-411	*Rpp1d* ^h^	IV	TAN	4.1	bcdefg	4.6	abcde
SB14	RN6-3-134211	*Rpp1b*	IV	TAN	4.3	abcde	4.4	cdefg
SB15	6108-1043321	*Rpp1c* ^h^	IV	TAN	4.4	abc	4.9	a
SB16	6116-5-4-1-1	Unknown	IV	TAN	4.1	cdefg	4.2	efghi
SB18	88-203-1-2-1	Unknown	V	TAN	4.3	abcde	4.8	ab
SB19	7088-198-212	Unknown	V	TAN	3.9	defghi	4.3	defghi
SB20	7088-198-521	Unknown	V	TAN	3.8	efghij	4.4	cdefg
SB21	6108-1662111	*Rpp1c* ^h^	V	TAN	4.0	cdefgh	4.7	abcd
SB22	6115-22-511	Unknown	V	TAN	3.5	ijklm	4.6	abcde
SB23	6113-216-121	*Rpp1e* ^h^	VI	TAN	3.9	defghij	4.8	ab
SB25	6106-132-1-2	Unknown	IV	TAN	3.9	defghi	4.2	efghi
SB30	Williams 82 ^i^	Susceptible	III	TAN	4.6	ab	4.8	ab
SB31	PI 594538A	*Rpp1b*	VIII	TAN	4.3	abcde	4.8	ab
SB33	PI 587886	*Rpp1d* ^h^	VI	TAN	4.3	abcd	4.0	ghij
SB34	PI 587880A	*Rpp1c* ^h^	VI	TAN	3.8	fghij	4.1	fghi
SB36	PI 587905	*Rpp1e* ^h^	VII	TAN	4.7	a	4.5	abcdef
SB37	PI 567099A	*rpp3*	VIII	TAN	3.1	mn	3.3	lm
SB43	PI 594723	Unknown	VII	TAN	3.2	lmn	3.8	ijk
SB44	PI 605779E	Unknown	VIII	TAN	3.8	efghij	3.8	ijk
SB47	PI 594760B	Unknown	VIII	TAN	4.2	bcdef	4.7	abcd
SB48	PI 567145C	Unknown	VIII	TAN	4.4	abc	4.7	abcd
SB49	Stafford ^i^	Susceptible	IV	TAN	3.9	defghij	4.3	cdefgh
SB50	5002T ^i^	Susceptible	V	TAN	4.3	abcde	4.8	ab
SB51	PI 605833	Unknown	IX	TAN	3.9	defghi	4.8	abc
SB52	Osage ^i^	Susceptible	V	TAN	3.7	ghijk	4.3	defghi
SB54	NC-Roy ^i^	Susceptible	VI	TAN	4.6	ab	4.2	efghi
SB55	PI 200456	*rpp5*	VIII	TAN	4.0	cdefgh	4.5	abcdef
Mean					3.5		3.6	

^a^ Maturity group; ^b^ RB: reddish-brown colored lesion, TAN: tan-colored lesions, NIM: near immune reaction with no macroscopically visible lesions; ^c^ Disease severity was determined based on lesion density (percentage of infected area) on the first trifoliate leaves. A five-point scale was used as previously described [29,30] where 1 = no visible lesions, 2 = a few lesions with approximately 1–20% of the area infected, 3 = lesion density with approximately 21–50% of the area infected, 4 = substantial lesion density with approximately 51–80% of the area infected, and 5 = very heavy lesion density with approximately 81–100% of the area infected; ^d^ Sporulation was rated based on the relative percentage of lesions producing urediniospores on each plant tested using a 1-to-5 scale where 1 = no sporulation, 2 = less than 25% sporulation, 3 = 26 to 50% sporulation, 4 = 51–75% sporulation, and 5 = 76 to 100% of the lesions sporulating [30,31]; ^e^ Resistant check; ^f^ Means followed by the same letter within a column and maturity group are not significantly different by the least significant difference test at *p* ≤ 0.05 as determined by the SAS GLMMIX procedure; ^g^ *Rpp2* and/or other gene(s); ^h^ Mapped to *Rpp1* but is not the same allele as *Rpp1b*; ^i^ Susceptible check.

## Data Availability

Relevant data generated or analyzed during this study are included in this article. Other data are available upon request from the corresponding author.
